# Psychosocial Mechanisms Linking the Social Environment to Mental Health in African Americans

**DOI:** 10.1371/journal.pone.0154035

**Published:** 2016-04-27

**Authors:** Scherezade K. Mama, Yisheng Li, Karen Basen-Engquist, Rebecca E. Lee, Deborah Thompson, David W. Wetter, Nga T. Nguyen, Lorraine R. Reitzel, Lorna H. McNeill

**Affiliations:** 1 Department of Kinesiology, College of Health and Human Development, The Pennsylvania State University, University Park, Pennsylvania, United States of America; 2 Department of Biostatistics, The University of Texas MD Anderson Cancer Center, Houston, Texas, United States of America; 3 Department of Behavioral Sciences, The University of Texas MD Anderson Cancer Center, Houston, Texas, United States of America; 4 College of Nursing and Health Innovation, Arizona State University, Phoenix, Arizona, United States of America; 5 USDA/ARS Children’s Nutrition Research Center, Baylor College of Medicine, Houston, Texas, United States of America; 6 Department of Psychology, Rice University, Houston, Texas, United States of America; 7 Department of Psychological, Health, and Learning Sciences, College of Education, University of Houston, Houston, Texas, United States of America; 8 Department of Health Disparities Research, The University of Texas MD Anderson Cancer Center, Houston, Texas, United States of America; Katholieke Universiteit Leuven, BELGIUM

## Abstract

Resource-poor social environments predict poor health, but the mechanisms and processes linking the social environment to psychological health and well-being remain unclear. This study explored psychosocial mediators of the association between the social environment and mental health in African American adults. African American men and women (*n* = 1467) completed questionnaires on the social environment, psychosocial factors (stress, depressive symptoms, and racial discrimination), and mental health. Multiple-mediator models were used to assess direct and indirect effects of the social environment on mental health. Low social status in the community (*p* < .001) and U.S. (*p* < .001) and low social support (*p* < .001) were associated with poor mental health. Psychosocial factors significantly jointly mediated the relationship between the social environment and mental health in multiple-mediator models. Low social status and social support were associated with greater perceived stress, depressive symptoms, and perceived racial discrimination, which were associated with poor mental health. Results suggest the relationship between the social environment and mental health is mediated by psychosocial factors and revealed potential mechanisms through which social status and social support influence the mental health of African American men and women. Findings from this study provide insight into the differential effects of stress, depression and discrimination on mental health. Ecological approaches that aim to improve the social environment and psychosocial mediators may enhance health-related quality of life and reduce health disparities in African Americans.

## Introduction

The social environment broadly influences health and health behaviors by shaping social norms, providing resources and opportunities for healthful behaviors, and buffering negative health outcomes [1]. Several theoretical frameworks and conceptual models posit direct and indirect associations between the social environment and physical and mental health [2–4]. Building on this work, Sorensen and colleagues proposed a conceptual model identifying social environmental factors of interest, including social status and social support, and suggested mechanisms through which these factors influence other social environmental factors, individual factors, and health [5].

Social status and social support are measures of the social environment that are directly associated with health behaviors [5]. Subjective social status describes an individual’s perception of their relative position in their community or in society [6]. Previous research has shown that subjective social status in the community and in society are distinct constructs and are independently associated with psychological functioning, health behaviors and physical health outcomes, such that poor/low social status is associated with poor health outcomes [6–11]. Moreover, subjective social status in the community and subjective social status in society, or in the United States, influence health outcomes through different pathways [8, 9]. Similarly, social support, defined as the resources provided by other people that can influence an individual’s ability to cope with stress [1, 12], has been associated with perceived health and mental health outcomes [13–16]. Although review studies have consistently shown that resource-poor social environments predict poor health [4, 17, 18], the mechanisms and processes linking the social environment to psychological health and well-being need further investigation [19].

Social ecological models and the conceptual model developed by Sorensen and colleagues suggest several psychosocial mechanisms and pathways through which the social environment influences health [5, 20]. One pathway, outlined by Berkman and Glass, involves influencing cognitive and emotional states, like perceived stress, depression, and discrimination [4]. Several recent studies have sought to understand the relationships among social support, social status, stress, depression, and racial discrimination [21–23] in various populations, including healthy adults and college students, cardiac patients, and cancer survivors [23–26]. These studies have shown modest mediation effects of cognitive and emotional states on the relationship between the social environment and health and have encouraged additional research to gain insight into the mechanisms underlying the risk associated with poor social support [26].

Although previous studies have looked at the mediated contributions of the social environment and general health, the contributions to mental health, specifically, are less understood. Limited or lower social support and perceived social standing may reduce the ability to cope with stressful situations and/or negatively influence the appraisal of certain situations in this population, leading to increased stress, depression, and perceived discrimination, which, in turn, lead to poorer mental health [2]. Few studies have explored the joint mediation effects of psychosocial factors on the relationship between the social environment and health, and none, to our knowledge, have reported the effects of racial discrimination as a psychosocial mediator of the relationship between the social environment and mental health. However, previous studies have found bivariate associations between the social environment and mental health and among social status, social support, perceived stress, depression and discrimination, providing justification for looking at these joint mediated effects in an effort to understand the relative importance and contribution of these psychosocial factors to mental health [21–26].

It is particularly important to look at these joint mediated effects among African American adults, who are underrepresented in the mental health literature. African Americans disproportionately experience stress, depression and discrimination and report a greater number of modifiable behavioral risk factors than non-Hispanic whites, putting them at greater risk of developing and dying from cancer and other chronic diseases [27]. In addition to increased physical disease burden, these risk factors are associated with poorer mental health and health-related quality of life [28], having a cyclic effect and further contributing to health disparities in this population. Thus, it is important to understand how these psychosocial factors jointly operate among African Americans and affect mental health.

This study sought to understand the underlying mechanisms through which the social environment influences mental health in African Americans. The primary aim of this study was to explore the potential psychosocial mediators of the relationship between the social environment and mental health in a large church-based sample of African American adults. We hypothesized that perceived stress, depressive symptoms, and perceived racial discrimination mediate the relationships between social environmental constructs—social status in the community, social status in the United States, and social support—and mental health.

## Materials and Methods

### Design

This study used baseline data from Project CHURCH (Creating a Higher Understanding of cancer Research and Community Health), a longitudinal cohort study examining the role of lifestyle/behavioral, social, and environmental factors on minority health and cancer-related disparities among a church-based sample of African Americans in Houston, Texas. The details of the study have been published previously [29–31]. For the present study, baseline questionnaire data were used to assess the effects of subjective social status, social support, perceived stress, depressive symptoms, and racial discrimination on mental health in adult African American men and women.

### Participants and Procedures

Project CHURCH study procedures and materials were reviewed and approved by the Institutional Review Board at The University of Texas MD Anderson Cancer Center, and participants provided written informed consent prior to completing any study activities. African American churchgoers (*n* = 1,467) were recruited to the study via flyers and printed media distributed at the church and in-person solicitation at church events. Eligible participants were at least 18 years of age, were able to read and speak English, lived in the Houston metropolitan area, had a valid home address and phone number, and attended church services. Participants completed an in-person baseline health assessment, which included measures of height and weight and a computer-based survey, between December 2008 and July 2009.

### Measures

#### Social Environment Constructs

Subjective social status was measured using the MacArthur Scale of Subjective Social Status [7], which has been found to be reliable and valid in racially/ethnically diverse populations [32]. We used two versions of the scale, each of which consists of a 10-rung ladder to represent where people believe they stand in their community (SSS-community) or in the United States (SSS-US), and participants select the rung that best represents where they stand relative to others [7]. The scale ranges from 1 to 10, and higher scores indicate higher status. These ladders have demonstrated adequate validity and reliability in studies with racially/ethnically and socioeconomically diverse participants, and the correlation between the SSS-community and SSS-US constructs was 0.517 (*p* < .001) in this sample.

Perceived social support was measured using the 12-item Interpersonal Support Evaluation List (ISEL-12) [33]. The ISEL-12 measures overall social support and three dimensions of social support—appraisal, belonging, and tangible support—and has been found valid and reliable for use in ethnic minority populations [34]. Cronbach’s alphas for ISEL-12 subscales ranged from 0.621 to 0.711 in this sample. Only overall social support was included in the current study. The scale ranges from 12 to 48, and a higher score indicates greater perceived social support.

#### Psychosocial Mediators

The four-item Perceived Stress Scale (PSS-4) was used to measure the degree to which situations are appraised as stressful [35]. The PSS-4 asks participants to indicate how often they felt or thought a certain way during the last month. The four-item scale has been found valid and reliable in ethnically diverse samples [36]. The scale ranges from 0 to 16, with higher scores indicating greater perceived stress. Cronbach’s alpha for the PSS-4 was 0.724 in this sample.

The 10-item Center for Epidemiological Studies Depression Scale (CES-D) was used to measure depressive symptoms [37]. The short form of the CES-D was developed to measure depressive symptoms in healthy populations [38] and has been found reliable and valid for detecting depressive symptoms in community-dwelling ethnic minorities [39, 40]. Participants were asked to indicate how they felt or behaved during the past week using a four-point scale from 0 to 3. The overall score is calculated by totaling the scores for the 10 items and ranges from 0 to 30. Higher scores indicated higher levels of depressive symptoms, and Cronbach’s alpha was 0.626 in this sample.

The Day-to-Day Unfair Treatment Scale, consisting of ten examples, was used to measure perceived racial discrimination [41]. Participants were asked to indicate on a four-point scale how often they experience these situations based on their race/ethnicity or skin color. Responses were reverse scored, and higher scores indicated greater perceived racial discrimination. Potential scores range from 10 to 40, and Cronbach’s alpha was 0.915 in this sample.

#### Mental Health

Mental health was measured using the Medical Outcomes Study’s 12-item Short-Form Survey (SF-12) version 2 [42]. The SF-12 was scored using Quality Metrics Health Outcomes Scoring Software 4.0, which provides a Mental Component Summary Score (MCS-12). The MCS-12 includes measures of vitality, social functioning, role limitations due to emotional problems, and mental health (psychological distress and psychological well-being; [43], providing a comprehensive picture of mental health and well-being. The SF-12 has been validated for use in African Americans [44]. In this study, MCS-12 scores ranged from 16 to 69 (Cronbach’s alpha = 0.843), and higher scores indicate better mental health.

#### Sociodemographics

Sociodemographics included gender, age, body mass index (BMI), education, annual household income, employment status, marital status, and number of children and were included as covariates in analyses.

### Statistical analysis

**Fig 1:**
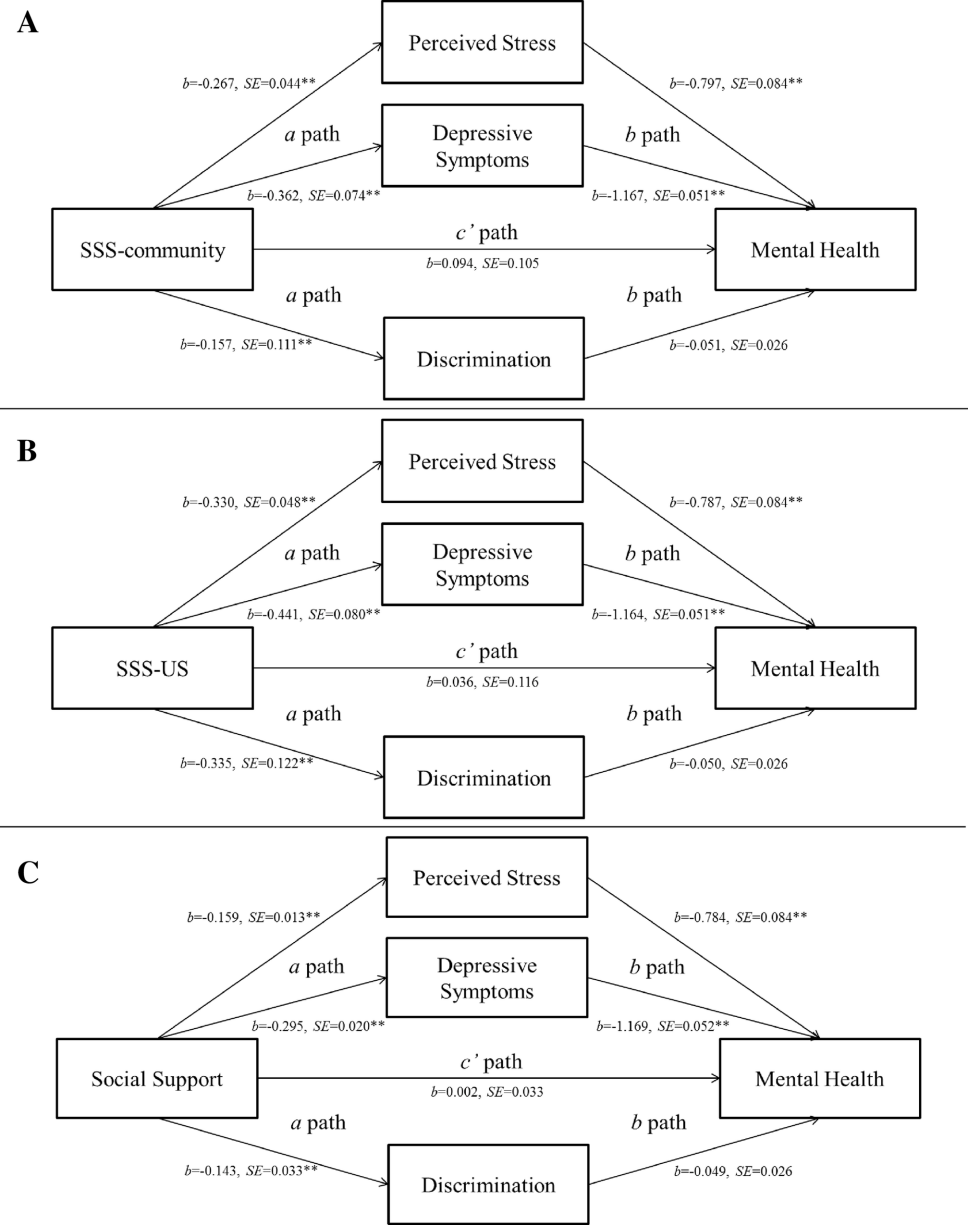
Direct Effects (*c*’ path) and Psychosocial Factor-Mediated Indirect Effects (*a* and *b* paths) of the Social Environment on Mental Health in African Americans. (A) Perceived stress and depressive symptoms, but not discrimination, jointly mediated the relationship between SSS-community and mental health (Effect = 0.643, *SE* = 0.128, 95% *CI*: 0.397, 0.891). (B) Perceived stress, depressive symptoms and discrimination jointly mediated the relationship between SSS-US and mental health (Effect = 0.790, *SE* = 0.140, 95% *CI*: 0.519, 1.068). (C) Perceived stress, depressive symptoms and discrimination jointly mediated the relationship between social support and mental health (Effect = 0.476, *SE* = 0.037, 95% *CI*: 0.403, 0.549). SE = standard error; SSS = subjective social status. *P < .05; **P < .01.

Descriptive statistics were calculated to explore participant characteristics for the total sample. Bivariate correlations, t-tests and analysis of variance were used to examine relationships among sociodemographics, subjective social status, social support, perceived stress, depressive symptoms, racial discrimination, and mental health. Mediation analyses consisted of three multiple-mediator models (Fig 1) to assess the indirect effects of subjective social status (SSS-community and SSS-US) and social support on mental health through perceived stress, depressive symptoms, and perceived racial discrimination. Each model was adjusted for gender, age, BMI, education, income, employment and marital status. Descriptive and preliminary analyses were completed in SPSS 22.0 (IBM SPSS Statistics, Armonk, NY), and mediation analyses were completed in SAS 9.3 (SAS Institute, Cary, NC) using version 2.041 of the PROCESS macro [45]. Indirect effects were tested using a non-parametric, bias-corrected bootstrapping procedure using 5,000 resamples from the data set [46]. Coefficients, standard errors (SE), and corresponding *p*-values were reported for *a*, *b*, and *c’* paths in multiple-mediator models; estimates, bootstrap SE, and corresponding bias-corrected bootstrap confidence intervals (CI) were reported for indirect effects [47]. The alpha level was set at two-sided 0.05 for all analyses, and the mediating effect was considered significant if the confidence interval of the indirect effect did not include the value zero [48].

## Results

### Participant Characteristics and Preliminary Analysis

Participants were generally middle-aged (mean = 45.2 years, standard deviation = 12.9). Most participants had completed some college or were college graduates (87.6%) and reported an annual household income ≥$40,000 (74.7%). Participant characteristics are listed in Table 1.

**Table 1:** Participant Characteristics and Scores (*n* = 1,467).

Characteristic or scale	Value
Characteristics [% (*n*)]	
Age [mean years (*SD*)]	45.2 (12.9)
BMI [mean kg/m^2^ (*SD)*]	31.6 (7.3)
Gender	
Male	25.4 (372)
Female	74.6 (1,095)
Education	
<Bachelor’s degree	51.6 (756)
Bachelor’s degree	29.5 (432)
>Bachelor’s degree	19.0 (278)
Annual household income	
<$40,000	25.3 (359)
$40,000–79,999	39.4 (559)
≥$80,000	35.3 (500)
Employed	73.9 (1,083)
Marital status	
Single/widowed/divorced	56.5 (827)
Married/living with partner	43.5 (638)
Children	
None	30.6 (449)
1 or more	69.4 (1,018)
Scales (scale range) [mean score (*SD*)]	
SSS-community (1–10)	7.3 (1.9)
SSS-US (1–10)	6.6 (1.7)
Social support (12–48)	41.1 (6.2)
Perceived stress (0–16)	4.6 (3.0)
Depressive symptoms (0–30)	5.8 (5.0)
Perceived racial discrimination (10–40)	20.5 (7.4)
Mental health (16–69)	50.3 (10.3)

SD = standard deviation; BMI = body mass index; SSS = subjective social status.

Gender, age, BMI, education, income, employment status, marital status, and number of children were significantly correlated with mental health outcomes (*p*’s<0.05; results not shown), and were controlled for in subsequent analyses. Correlations among social status and social support variables ranged from 0.160 to 0.199 (*p*’s<0.001), and correlations among psychosocial mediators ranged from 0.159 to 0.678 (*p*’s<0.001). Social status (SSS-community: *r* = 0.137, *p*<0.001; SSS-US: *r* = .164, *p*<0.001), social support (*r* = 0.278, *p*<0.001), perceived stress (*r* = -0.619, *p*<0.001), depressive symptoms (*r* = -0.720, *p*<0.001), and racial discrimination (*r* = -0.175, *p*<0.001) were significantly correlated with mental health.

### Total Effects

After adjusting for gender, age, BMI, education, income, employment and marital status, and number of children, SSS-community (*b* = 0.549, *SE* = 0.154, *p*<0.001) and SSS-US (*b* = 0.826, *SE* = 0.168, *p*<0.001) had significant total effects on mental health and accounted for 5.6% and 6.4% of the variance in mental health, respectively. Overall social support also had a significant total effect on mental health (*b* = 0.478, *SE* = 0.044, *p*<0.001), accounting for 12.4% of the variance in mental health. Thus, higher SSS-community, higher SSS-US, and greater social support were associated with better mental health.

### Multiple-Mediator Models

Perceived stress, depressive symptoms, and racial discrimination significantly mediated the effect of the social environment on mental health in single-mediator models (results not shown). All three psychosocial mediators were therefore included in multiple-mediator models for mental health (Fig 1), and the indirect effects of each mediator, controlling for the effect of the other two mediators, and the joint indirect effects for all mediators are shown in Table 2. Perceived stress and depressive symptoms, but not racial discrimination, jointly mediated the effect of SSS-community on mental health, and perceived stress, depressive symptoms, and perceived racial discrimination jointly mediated the effect of SSS-US and social support on mental health. Lower subjective social status and social support were associated with greater perceived stress, depressive symptoms, and perceived racial discrimination, which were associated with poorer mental health. Together, these psychosocial mediators explained 44.6–51.4% of the variance in mental health in addition to the 5.6–12.4% explained by social environmental variables above.

**Table 2:** Indirect Effects on Mental Health Outcomes in Multiple-Mediator Models.

Proposed mediator	Mental health		
	Effect	*SE*	95% *CI*
SSS-community			
Perceived stress	0.213	0.045	0.133, 0.311
Depressive symptoms	0.422	0.099	0.235, 0.622
Discrimination	0.008	0.008	-0.001, 0.030
Total	0.643	0.128	0.397, 0.891
SSS-US			
Perceived stress	0.259	0.051	0.167, 0.367
Depressive symptoms	0.513	0.108	0.307, 0.728
Discrimination	0.017	0.011	0.001, 0.049
Total	0.790	0.140	0.519, 1.068
Social support			
Perceived stress	0.124	0.017	0.095, 0.160
Depressive symptoms	0.345	0.033	0.281, 0.411
Discrimination	0.007	0.004	0.000, 0.018
Total	0.476	0.037	0.403, 0.549

SE = standard error; CI = confidence interval.

## Discussion

In this study, we sought to understand the underlying mechanisms through which the social environment influences mental health in African Americans and explored the effects of multiple psychosocial mediators on the associations of subjective social status and social support with mental health in African Americans. The results revealed potential mechanisms through which subjective social status and social support influence the mental health of African American men and women, and showed that greater subjective social status and social support were directly and positively associated with better mental health. Perceived stress, depressive symptoms, and racial discrimination jointly mediated the relationships between social environment constructs and mental health and accounted for approximately 50% of the variance in mental health.

Although the relationship between greater social support and better health outcomes has been established by researchers [4], our study is one of the first to demonstrate the mediating effects of specific psychosocial factors on this relationship in African Americans and provides important insight into how these factors act as chronic stressors and jointly contribute to disparities in mental health [49, 50]. Previous studies exploring chronic stressors in African Americans have focused on racial discrimination and have postulated coping mechanisms that African Americans use in response to racial discrimination that have a detrimental effect on health [51, 52]. One such coping style is John Henryism, which describes the predisposition of African Americans to actively cope with psychosocial and environmental stressors, including perceived stress, depressive symptoms and racial discrimination, at the detriment of their health [53]. However, previous studies exploring the John Henryism hypothesis have focused on the impact of racial discrimination and have not explored the contributions of multiple psychosocial factors on the mental health of African Americans. In the current study, we found that depressive symptoms accounted for a greater proportion (64.9–72.5%) of the association between the social environment and mental health than stress and perceived racial discrimination, giving us insight into the differential effects of these psychosocial stressors on mental health.

Findings from this study increase our understanding of what is driving the relationship between psychosocial stressors and mental health in African Americans and echo previous research stressing the importance of taking into account the social context in addition to the economic context [53–55]. In our sample of educated and high income men and women, we found that these chronic psychosocial stressors, particularly depressive symptoms, continue to impact the health and well-being of African Americans, deemphasizing the role of the socioeconomic position. Thus, ecological approaches that aim to systematically modify both the social environment and psychosocial mediators, including perceived stress, depression, and perceived racial discrimination, may have the greatest impact on mental health in African Americans [4, 54], and may lead to additional improvements in psychological factors, health behaviors, and physical health [4].

This study had several notable strengths and limitations. We explored the joint effects of multiple psychosocial mediators on the associations of subjective social status and social support with health in a large sample of African Americans. The linkages between the social environment and stress, depression, and perceived racial discrimination and between these psychosocial factors and mental health have been shown repeatedly but almost always in separate studies [56]. Another strength of this study was the use of multiple measures of the social environment, including social support, social status in the community, and social status in the U.S. Although these measures behaved similarly among African Americans adults in the current study, previous studies have shown that social status in the community and in the U.S. are distinct constructs and influence mental health differently in diverse populations [8, 9].

Limitations of the current study include its cross-sectional design, limiting causal inferences. Although previous research supports the pathways examined in the current study [19, 57–59], the potential for reverse causation and other mediational pathways cannot be ruled out and warrants further research. The use of self-reported measures of stress and health may contribute to response bias and limits interpretation of findings. Objective measures of stress and health, including biomarkers and cardiometabolic risk factors, such as cortisol levels, blood pressure, and cholesterol levels, are needed to confirm our findings. Additional study limitations include the use of an educated, predominantly female sample of African American churchgoers from a large urban city, which may have contributed to higher self-reported social support and coping strategies. Although results may not be generalizable to other samples, including rural populations or those of low socioeconomic status, they are applicable to the vast majority of African Americans (82%) and those in the South (81%) who report a formal religious affiliation [60]. Future studies will need to include a larger proportion of men and should explore other psychosocial mechanisms (e.g. negative affect, loneliness, and anxiety).

Our findings provide important evidence for the link between the social environment and mental health in African Americans and add to our understanding of the mental health consequences of chronic psychosocial stressors in light of social resources in this population. African Americans continue to disproportionately suffer from chronic diseases due to disparities in risk factors and health behaviors. Our results highlight the need to address psychosocial factors as part of chronic-disease prevention programs and interventions in an effort to improve mental health and reduce health disparities among African Americans.
